# Escape of Hepatitis C Virus from Epitope I Neutralization Increases Sensitivity of Other Neutralization Epitopes

**DOI:** 10.1128/JVI.02066-17

**Published:** 2018-04-13

**Authors:** Jun Gu, Joshua Hardy, Irene Boo, Patricia Vietheer, Kathleen McCaffrey, Yousef Alhammad, Abha Chopra, Silvana Gaudieri, Pantelis Poumbourios, Fasséli Coulibaly, Heidi E. Drummer

**Affiliations:** a Virus Entry and Vaccines Laboratory, Life Sciences, Burnet Institute, Melbourne, Australia; b Department of Microbiology, Faculty of Medicine, Nursing and Health Sciences, Monash University, Clayton, Australia; c Monash Biomedicine Discovery Institute, Department of Biochemistry and Molecular Biology, Monash University, Clayton, Australia; d Department of Microbiology and Immunology at the Peter Doherty Institute, The University of Melbourne, Melbourne, Australia; e Institute for Immunology and Infectious Diseases, Murdoch University, Murdoch, Australia; f School of Human Sciences, University of Western Australia, Nedlands, Australia; g Division of Infectious Diseases, Vanderbilt University Medical Center, Nashville, Tennessee, USA; Washington University School of Medicine

**Keywords:** glycoproteins, hepatitis C virus, neutralizing antibodies, vaccines

## Abstract

The hepatitis C virus (HCV) E2 glycoprotein is a major target of the neutralizing antibody (nAb) response, with multiple type-specific and broadly neutralizing antibody (bnAb) epitopes identified. The 412-to-423 region can generate bnAbs that block interaction with the cell surface receptor CD81, with activity toward multiple HCV genotypes. In this study, we reveal the structure of rodent monoclonal antibody 24 (MAb24) with an extensive contact area toward a peptide spanning the 412-to-423 region. The crystal structure of the MAb24–peptide 412-to-423 complex reveals the paratope bound to a peptide hairpin highly similar to that observed with human MAb HCV1 and rodent MAb AP33, but with a different angle of approach. In viral outgrowth experiments, we demonstrated three distinct genotype 2a viral populations that acquired resistance to MAb24 via N415D, N417S, and N415D/H386R mutations. Importantly, the MAb24-resistant viruses exhibited significant increases in sensitivity to the majority of bnAbs directed to epitopes within the 412-to-423 region and in additional antigenic determinants located within E2 and the E1E2 complex. This study suggests that modification of N415 causes a global change in glycoprotein structure that increases its vulnerability to neutralization by other antibodies. This finding suggests that in the context of an antibody response to viral infection, acquisition of escape mutations in the 412-to-423 region renders the virus more susceptible to neutralization by other specificities of nAbs, effectively reducing the immunological fitness of the virus. A vaccine for HCV that generates polyspecific humoral immunity with specificity for the 412-to-423 region and at least one other region of E2 is desirable.

**IMPORTANCE** Understanding how antibodies neutralize hepatitis C virus (HCV) is essential for vaccine development. This study reveals for the first time that when HCV develops resistance to a major class of bnAbs targeting the 412-to-423 region of E2, this results in a concomitant increase in sensitivity to neutralization by a majority of other bnAb specificities. Vaccines for the prevention of HCV infection should therefore generate bnAbs directed toward the 412-to-423 region of E2 and additional bnAb epitopes within the viral glycoproteins.

## INTRODUCTION

Hepatitis C virus (HCV) infects 60 to 120 million people worldwide and causes chronic liver disease and hepatocellular carcinoma (1). HCV is an RNA virus of the genus Hepacivirus of the Flaviviridae family and displays a high degree of genetic and antigenic variability. As a result, HCV is classified into seven distinct genotypes that differ by up to 30% at the nucleotide level and 67 confirmed subtypes that differ by up to 20% at the nucleotide level (2). In addition, in infected people, the low-fidelity RNA-dependent RNA polymerase creates HCV quasispecies (3) that are under selection pressure via major histocompatibility complex restriction, T cell and antibody recognition, or antiviral treatment (4).

Entry of HCV into hepatocytes is mediated by viral glycoproteins E1 and E2, which form heterodimers on the surface of the virions. The binding of E2 to host cell receptor CD81 is an essential step in HCV entry; thus, HCV E2 is a major target of neutralizing antibodies (nAbs). A recombinant form of E2 containing the N-terminal portion spanning residues 384 to 661 can be expressed independently of the remaining glycoprotein, resulting in the secretion of a receptor-binding domain (RBD) that retains CD81-binding and key neutralization epitopes (5–7). Within the RBD are three variable regions, hypervariable region 1 (HVR1; residues 384 to 410), HVR2 (residues 460 to 485), and the intergenotypic variable region (igVR/VR3; residues 570 to 580).

Glycoprotein E2 is a target for the generation of nAbs. Two independently derived core domain structures of HCV E2 show a central immunoglobulin-like β-sandwich flanked by front and back layers (8, 9). These E2 core domain structures lack three-dimensional (3D) information for the N-terminal region (residues 384 to 419) that includes HVR1; residues 452 to 492, which include HVR2; and the C-terminal region beyond residue 645 (8). The contact area for interaction with CD81 and a number of broadly neutralizing monoclonal antibodies (bnMAbs) reside on the so-called neutralizing face of E2, with HVR2 and the igVR located on the opposite “nonneutralizing” face. HVR1 is immunodominant in natural infection, and antibodies directed to this epitope can mediate the neutralization of autologous viral isolates and rapidly select escape variants. Three additional regions within E2 have been identified as targets of bnAbs and overlap regions involved in CD81 interactions, i.e., residues 412 to 423 (domain E, epitope I, AS412), residues 434 to 446 (domain D, epitope II, AS434), and antigenic region 3 (AR3), which comprises the entire front or neutralizing face of E2. In addition, human bnMAbs have been isolated that are specific for epitopes that comprise the E1E2 heterodimer and are referred to as AR4 and AR5 (10).

bnAbs toward the 412-to-423 region are infrequently elicited in natural HCV infection, being detected in only 2.5 to 15% of chronically infected people (11, 12). Within this region, two amino acid residues, W420 and H421, are important for the binding of E2 to CD81 (13, 14). Several bnMAbs targeting this region have been isolated from rodents vaccinated with E2 antigens and from humans through natural infection, including mouse AP33 (15–17), rat 3/11 (17, 18), human HCV1 and 95-2 (19), human HC33.1 and related antibodies (12), H77.39 (20), MRCT10.v362 and hu5B3.v3 (21), and MAb24 (7). As the 412-to-419 region is disordered in E2 core structures, structural information on epitope I has only been obtained by using antibodies in complex with synthetic peptides spanning the 412-to-423 region. These studies have revealed alternate conformations of bnMAb-bound peptide: a β-hairpin structure (AP33, HCV1, MRCT10.v362, and hu5B3.v3), an extended structure (3/11), and a largely extended structure containing a short three-residue β-strand (HC33.1) (21–26). In addition, the angle of approach is distinct, even for MAbs that bind their epitopes in very similar conformations. The description of alternate conformations of the 412-to-423 region may suggest that it displays conformational flexibility and could represent another mechanism of immune evasion (22).

A vaccine that prevents new HCV infections is required to support disease elimination efforts. Such a vaccine ideally should generate bnAbs able to prevent infection by all circulating genotypes and subtypes of HCV. Antibody specifcities targeting domain E/epitope I (HCV1-like) are highly desirable components of the vaccinal immune response. We showed previously that immunization of small animals with an RBD construct lacking the three variable regions (Δ123) led to the generation of domain E/epitope I-targeted bnAbs that were effective against the six major HCV genotypes. It is therefore essential to understand how HCV could escape domain E/epitope I-targeting antibodies and the overall effect of escape mutations on virus fitness. Previously, we described domain E/epitope I-specific murine monoclonal antibody 24 (MAb24), which was isolated from a mouse immunized with Δ123 from genotype 1a H77c (7). The epitope of MAb24 is located in the 412-to-423 region of E2, and MAb24 prevents interaction with CD81 and has broad neutralization properties (7).

In this study, we further characterized the neutralization mechanism of MAb24 by examining the dynamic evolution of HCV quasispecies (genotype 2a) *in vitro* under the extreme selective pressure of MAb24 by the use of next-generation sequencing analysis. We found that resistance to MAb24 neutralization involves either a direct mutation in its epitope (N415D) or a shift in glycosylation (N417S) that obstructs binding by MAb24. In addition, we identify an additional mutation in HVR1 (H386R) that improves the fitness of viruses containing the N415D mutation. Structural analyses showed that an epitope I peptide bound to MAb24 adopts a β-loop conformation but with an angle of approach different from that of previously described antibodies. We show that acquisition of these mutations results in viruses that are resistant to MAb24 and HCV1. However, viruses containing N415D, N417S, or H386R/N415D have enhanced sensitivity to neutralization by domain E/epitope I-specific HC33.1 and other bnMAbs targeting epitopes outside this domain. Our findings suggest that acquisition of mutation(s) N415D, N417S, or H386R/N415D does not confer fitness on HCV in the context of a nAb response during infection. This study reinforces the notion that domain E/epitope I specificities are highly desirable components of the vaccinal immune response to HCV.

## RESULTS

### HCV replication under the selective pressure of MAb24.

**FIG 1:**
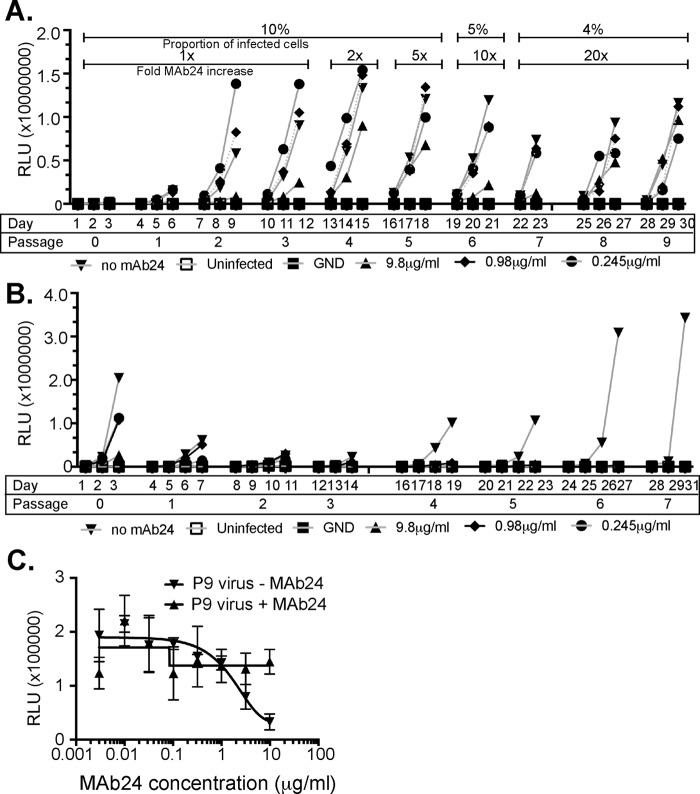
Generation of genotype 2a HCVcc MAb24 neutralization resistance variants. (A) At day 1, HCVcc, produced by transfection of Huh7.5 cells, was incubated in the presence of MAb24. At day 3, cell-free virus, together with infected cells from the passage, was added to naive Huh7.5 cell monolayers in the presence of MAb24 and repeated every 3 days for a total of nine passages. The amount of MAb24 was progressively increased, and the proportion of infected cells was gradually decreased at the passages indicated, as shown at the top. Luciferase activity in the supernatant fluid (in relative light units [RLU]) was measured on the days indicated. (B) At day 1, HCVcc, produced by transfection of Huh7.5 cells, was incubated in the presence of MAb24, and at day 3, tissue culture supernatant fluid containing cell-free virus was applied to naive Huh7.5 monolayers in the presence of MAb24; this was repeated for a total of seven passages, and the RLU in the supernatant fluid were measured on the days indicated. (C) Virus harvested at passage 9 (P9) from panel A cultured at a starting MAb24 concentration of 9.8 μg/ml or cultured in the absence of MAb24 was incubated with serial dilutions of MAb24 and added to naive Huh7.5 cells. Infectivity was measured 72 h later. GND is a mutation in NS5B that renders HCVcc replication incompetent.

To determine whether HCV is able to escape MAb24 neutralization, extracellular G2a cell culture-produced HCV (HCVcc), derived from transfection of cells with RNA of the wild-type (WT) JC1flag2 (p7-NS-GLUC2A) virus (JC1flag), was mixed with 0, 0.245, 0.98, or 9.8 μg/ml MAb24, which corresponds to the antibody concentration required to effect 0, 50, 70, or 90% neutralization of the original viral stock, respectively, prior to the inoculation of Huh7.5 cells. The inoculated cells were cultured for 3 days in the presence of MAb24 prior to passaging on naive Huh7.5 cells. To maximize the chance of selecting diverse escape mutants, infected cells from each passage were included with corresponding culture supernatants in the subsequent passage. The amount of MAb24 added to cultures was progressively increased from passage 4 onward such that 2 times (passage 4), 5 times (passage 5), 10 times (passage 6), or 20 times (passages 7 to 9) the original amount of MAb24 was used. Within 27 days of culture, virus replication was detected at MAb24 concentrations 20 times the 90% inhibitory concentration (IC_90_), suggesting that highly resistant viruses had been selected (Fig. 1A). Replicating virus was not recovered in a parallel passaging experiment where only tissue culture fluid containing extracellular virus was sequentially passaged on naive Huh7.5 cells in the presence of the concentrations of MAb24 described above, despite lengthening of the passage interval from 3 to 4 days (Fig. 1B). We confirmed that the passage 9 virus stock obtained as shown in Fig. 1A was able to infect naive Huh7.5 cells and replicate in the absence of MAb24. Neutralization assays confirmed that the passage 9 virus stock is resistant to MAb24, whereas the virus recovered at passage 9 passaged in the absence of MAb24 remained sensitive to neutralization by MAb24 (Fig. 1C). Together, these findings indicate that HCV is able to generate MAb24-resistant variants within 27 days *in vitro*.

### Characterization of MAb24-resistant quasispecies.

**FIG 2:**
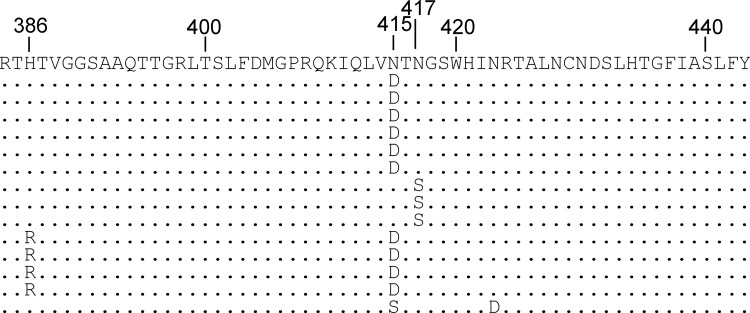
Sanger sequencing of cDNA clones obtained following the generation of MAb24-resistant virus. cDNA was prepared from viral RNA present in the supernatant fluid of infected cells at passage 9 and sequenced with BigDye Terminator. The entire E1E2 region was sequenced, and the region where mutations were detected is shown (residues 384 to 443).

To identify mutations associated with resistance to MAb24 neutralization in the infected cell passaging experiment, cDNA was prepared from passage 9 extracellular HCVcc where the starting (time zero) concentration of MAb24 was 9.8 μg/ml. Sanger sequencing of the complete E1E2 region showed that single adaptive mutations accumulated in the E2 epitope I region (residues 412 to 423), at position N415 (43%) or N417 (21%). In addition, 29% of the clones isolated contained both N415D and an additional mutation in HVR1, H386R (Fig. 2). Dominant mutations were not found in E1 or elsewhere in E2 by this method (not shown). These mutations were not present in cDNA prepared from HCVcc isolated after nine passages in the absence of MAb24 (data not shown), indicating that they arose because of MAb24 selection.

Illumina high-throughput sequencing was used to further examine the *in vitro* evolution of HCVcc toward MAb24 resistance. Barcoded primers were used to sequence cDNA prepared from cell-free HCVcc and HCVcc extracted from cell lysates, targeting the region encompassing amino acids 410 to 550, which includes epitope I. Group-wide comparison of virus sequences obtained at different time points and in the presence of MAb24 showed single nucleotide polymorphisms (SNPs) generating mutations at amino acid residues N415 and N417, consistent with the Sanger sequencing results (Table 1). N415D was the most common mutation observed; it was found in 27% of the population at passage 7 and in 79% of the population at passage 9. Other variants at the N415 position were also detected at passages 7 and 9, including N415S, N415T, and N415G at a low frequency (≤1%). In addition, N417S was detected at a very low frequency (<1%) at passage 4, in 1% of the sequences at passage 7, and in 12% of the sequences at passage 9. Traces of variants at V414 and G418 were also found at passage 7, but they remained at an extremely low frequency at passages 7 to 9. Additional variants external to the MAb24 epitope were transiently observed at a frequency of <1% at I422, D431, I438, Y443, M456, E482, N534, and L541 in sequences obtained between passages 4 and 7. Variants with P, V, and I at position L540 were observed at a low frequency (≤1%) at passage 4 but did not increase in frequency over subsequent passages. Greater than 99% of the sequences of HCVcc isolated in the absence of MAb24 were those of the parental virus, confirming that mutations at positions N415 and N417 are selected by MAb24 (data not shown).

**TABLE 1:** Frequencies of mutations in cell-free virus within the E2 region

WT residue	Mutation(s) (frequency [%]) at passage:^*a*^			
	0	4	7	9
V414	V (100)	V (100)	V (99), A (<1)	V (99), A (<1)
N415	N (100)	N (100)	N (70), D (27), S (1), T (<1), G (<1)	D (79), N (16), S (3), T (<1), G (<1)
N417	N (100)	N (99), S (<1)	N (98), S (1)	N (87), S (12)
G418	G (100)	G (100)	G (99), A (<1)	G (99), A (<1)
I422	I (100)	I (100)	I (100)	I (99), V (<1)
D431	D (100)	D (100)	D (99), G (<1)	D (100)
I438	I (100)	I (100)	I (100)	I (99), V (<1)
Y443	Y (100)	Y (99), H (<1)	Y (100)	Y (100)
F447	F (100)	F (100)	F (100)	F (99), L (<1)
M456	M (100)	M (99), V (<1)	M (100)	M (100)
E482	E (100)	E (100)	E (100)	E (99), G (<1)
N534	N (100)	N (100)	N (99), S (<1)	N (99), D (<1)
L540	L (100)	L (98), P (1), V (<1), I (<1)	L (99), P (<1), V (<1)	L (99), P (<1), V (<1)
L541	L (100)	L (100)	L (99), S (<1)	L (100)

a Extracellular virus was obtained from the selected passage numbers from cultures of Huh7.5 cells initially infected with virus in the presence of 9.8 μg/ml MAb24 with serial passage of infected cells and extracellular virus.

### Characterization of viruses with N415D, N417S, and H386R/N415D mutations.

**FIG 3:**
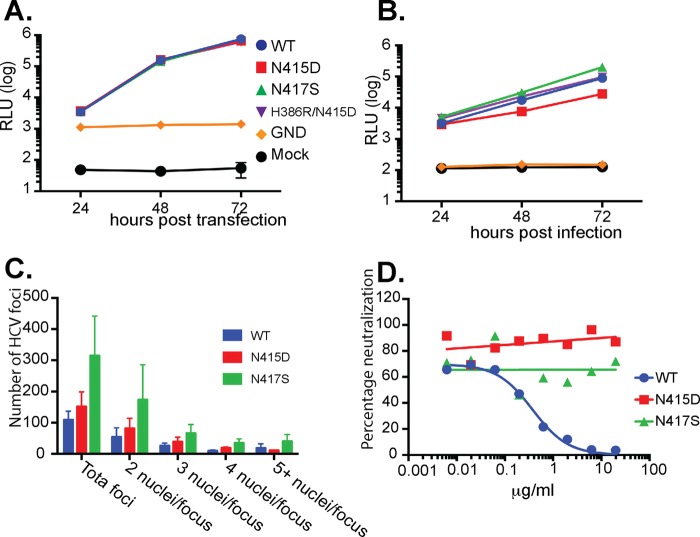
Viruses with N415D, N417S, and N415D/H386R mutations are replication competent, infectious, and resistant to MAb24 neutralization. (A) RNA was prepared from reverse-engineered HCVcc containing mutations Asp415, Ser417, and Arg386/Asp415 and transfected into Huh7.5 cells. Luciferase activity was measured in the supernatant fluid every 24 h. RLU, relative light units. (B) Supernatant fluid was collected 72 h after RNA transfection into Huh7.5 cells, normalized for infectivity, and used to infect naive Huh7.5 cell monolayers. Luciferase activity was measured in the supernatant fluid every 24 h. (C) Normalized amounts of HCVcc were added to naive Huh7.5 cells. After 72 h, monolayers were fixed and stained with antibody to NS5A and counterstained with propidium iodide and the number of nuclei per focus was determined. Data are representative of two independent experiments performed in duplicate. (D) Reverse-engineered viruses with N415D and N417S were incubated with serial dilutions of MAb24 and applied to naive Huh7.5 cell monolayers. Luciferase activity in the supernatant fluid was measured at 72 h postinfection. Graphs were drawn in Prism v7 by using nonlinear regression.

To further examine the effects of MAb24 resistance-conferring mutations on viral fitness, the three most frequently occurring mutations in the viral quasispecies that exhibited high levels of resistance to MAb24, N415D, N417S, and H386R/N415D, were each reverse engineered into the parental JC1flag G2a genome. The replication and infectivity of the three mutant viruses were characterized and compared with those of the WT virus. All three viral mutants replicated at WT levels in cells transfected with the viral genomic RNA (Fig. 3A). When the infectivity of the resultant cell-free viruses was examined, small differences between the three mutants and the parental virus were observed. The infectivity of HCVcc carrying the N415D mutation was reduced ∼6-fold but was restored 3-fold when the H386R mutation was included. In contrast, the N417S mutant virus was at least as infectious as the parental virus (Fig. 3B). These data suggest that N415D is associated with a fitness cost but fitness is partially restored by the addition of H386R, whereas no fitness cost is associated with the acquisition of N417S. The differences in fitness between the N415D and N417S mutant viruses were further examined by using a fluorescent-focus assay where the total number of NS5A-positive foci and the number of positive foci containing two or more nuclei were enumerated 72 h postinfection with normalized amounts of viral inoculum. The results (Fig. 3C) reveal that the N417S mutant is approximately 2-fold more infectious that the WT and the N415D mutant and is likely to spread faster between apposed cells, as evidenced by the greater proportion of foci containing two or more nuclei relative to the WT or the N415D mutant. To confirm that the epitope I mutations are directly associated with viral resistance to an epitope I-specific antibody, we examined their MAb24 neutralization profiles. While the WT virus was readily neutralized by MAb24, virus containing either N415D or N417S exhibited resistance to neutralization by MAb24, recapitulating the phenotype observed at passage 9 (Fig. 3D).

### Mutations conferring resistance to MAb24 result in an increase in sensitivity to neutralization by other MAbs.

**FIG 4:**
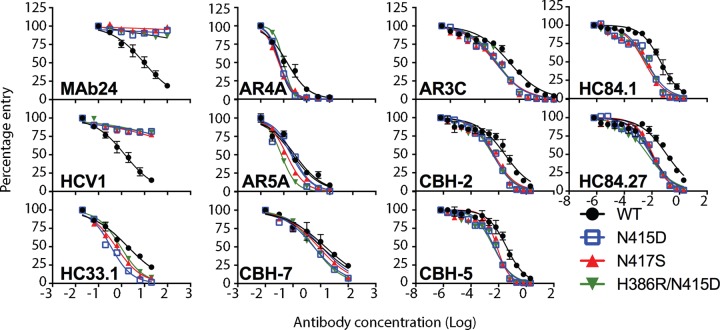
Neutralization of HCVcc with N415D, N417S, and H386R/N415D mutations by human MAbs. Serial dilutions of each MAb were incubated with parental HCVcc (WT) or N415D, N417S, or H386R/N415D mutant virus. After 72 h, the luciferase activity was measured. Data shown are the mean of at least three independent experiments performed in triplicate, with the exception of AR5A, which was performed once in triplicate. Neutralization curves were drawn by nonlinear regression analysis (Prism v7) and used to obtain IC_50_s (Table 2).

To investigate whether mutations at H386, N415, and N417 affected neutralization by other bnMAbs specific to this region and MAbs whose epitopes lie elsewhere in E2, we performed neutralization assays with previously well-characterized human bnMAbs. We first examined neutralization by other bnMAbs that also recognize epitopes in the 412-to-423 region (HCV1, HC33.1). Genotype 2a HCVcc with N415D, N417S, or the H386R/N415D double mutation is resistant to neutralization by HCV1, which recognizes its epitope within the 412-to-423 region in a hairpin conformation (24). HC33.1 is a bnMAb whose linear epitope is also contained within the 412-to-423 region. However, an extended conformation of this epitope is observed when it is bound to HC33.1 (25). In contrast to the data obtained with MAb24 and HCV1, N415D, N417S, and H386R/N415D mutant viruses were significantly more sensitive to HC33.1 neutralization than the WT was (Fig. 4 and Table 2).

**TABLE 2:**
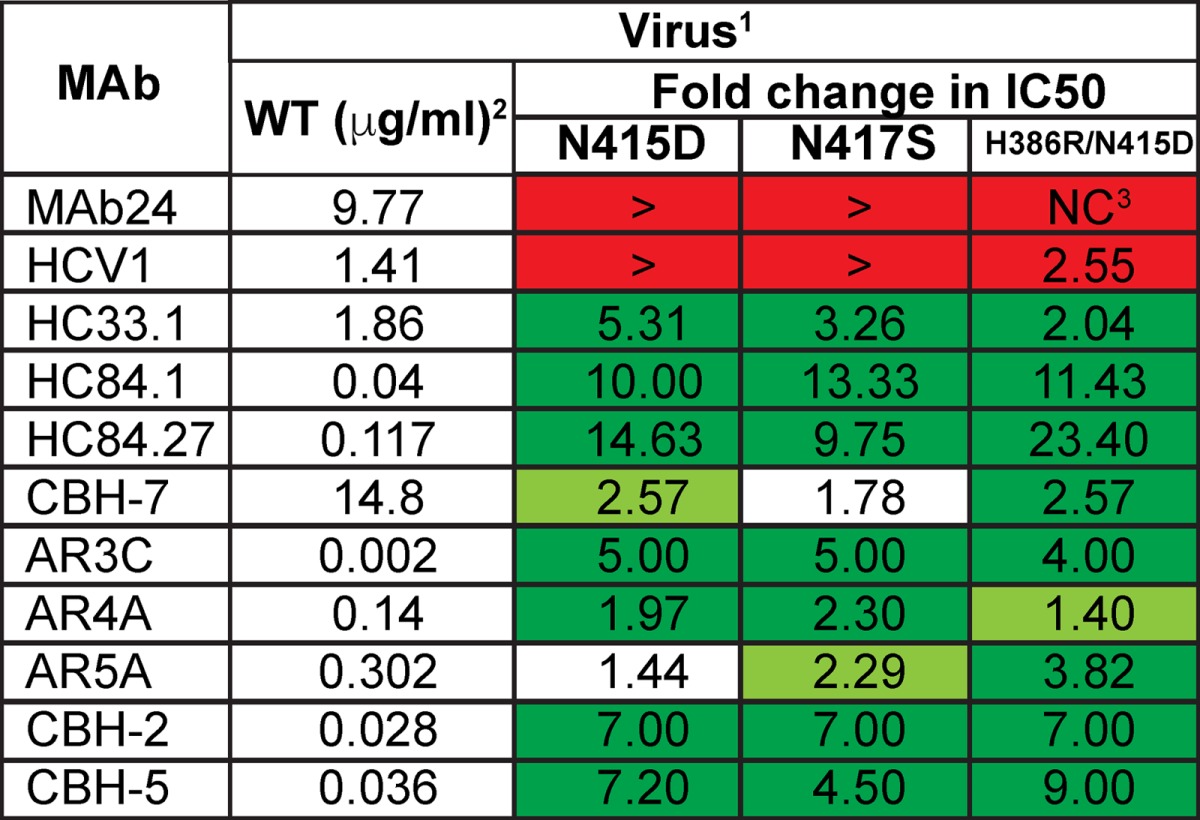
Susceptibility of HCV to neutralization by MAbs^*a*^

a (1) Data are the mean values of at least three experiments calculated from the data shown in Fig. 4 by using the log inhibitor versus normalized response curve in Prism v7.0. The symbol > indicates that the amount of antibody required to inhibit entry is greater than the highest concentration tested. Red indicates significantly more resistance than the parental WT virus (*P* < 0.001). Dark green and light green indicate significantly more sensitivity to neutralization than the parental WT virus (*P* < 0.001 and *P* = 0.004 to 0.007, respectively). White indicates no significant difference from the parental WT virus (*P* > 0.05). (2) The IC_50_ (μg/ml) of each MAb for the WT virus and fold changes in IC_50_ relative to the WT virus are shown. (3) NC, not converged (no *P* value calculated). Red, fold increase in resistance; green and white, fold increase in sensitivity.

The HCVcc variants were next tested with a panel of E2-specific human bnMAbs that are directed to epitopes outside the 412-to-423 region. The bnMAb panel included HC84.1 and HC84.27 directed to the 430-to-451 region (domain D, epitope II, or AS434) (27) and bnMAbs that bind discontinuous sequences in domain B (CBH-2, epitope contains CD81 binding loop residues 519 to 535, CBH-5), domain C (CBH-7, epitope includes N540 and W549) (28, 29), AR3 (AR3C), AR4 (AR4A), and AR5 (AR5A) (10). In almost all cases, N415D, N415D/H386R, and N417S mutant viruses were significantly more sensitive to neutralization by these bnMAbs than the parental (WT) virus was. The exceptions were the N417S mutant with CBH-7 and the N415D mutant with AR5A. These results suggest that the acquisition of resistance to MAb24 via mutations in the 412-to-423 region results in a global shift in neutralization sensitivity toward other bnMAbs recognizing distinct epitopes in E1E2.

### E2 epitope I adopts a β-hairpin conformation when bound to MAb24.

**FIG 5:**
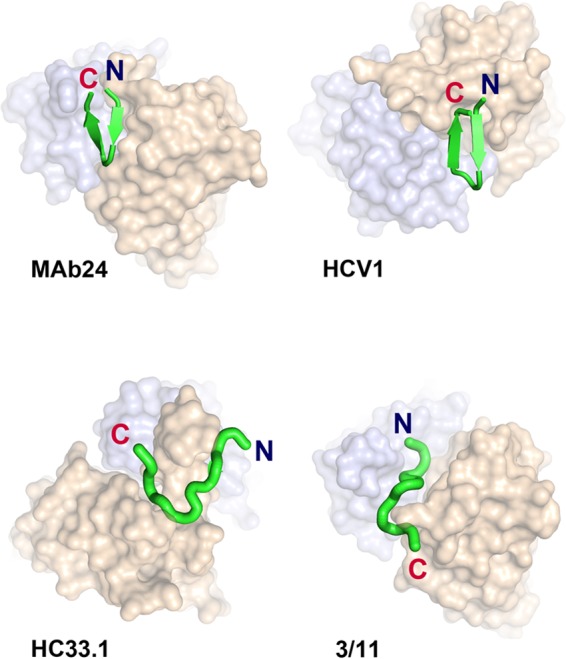
Epitope 1, represented as a green ribbon, adopts a β-hairpin conformation when bound to MAb24 and HCV1 (PDB code 4DGY), in contrast to the extended conformations found in structures of HC33.1 (PDB code 4XVJ) and 3/11 (PDB code 4WHT). Note the difference in the angles of approach between MAb24 and HCV1 evident from the shift of the heavy (brown) and light (blue) chains in this orientation.

**FIG 6:**
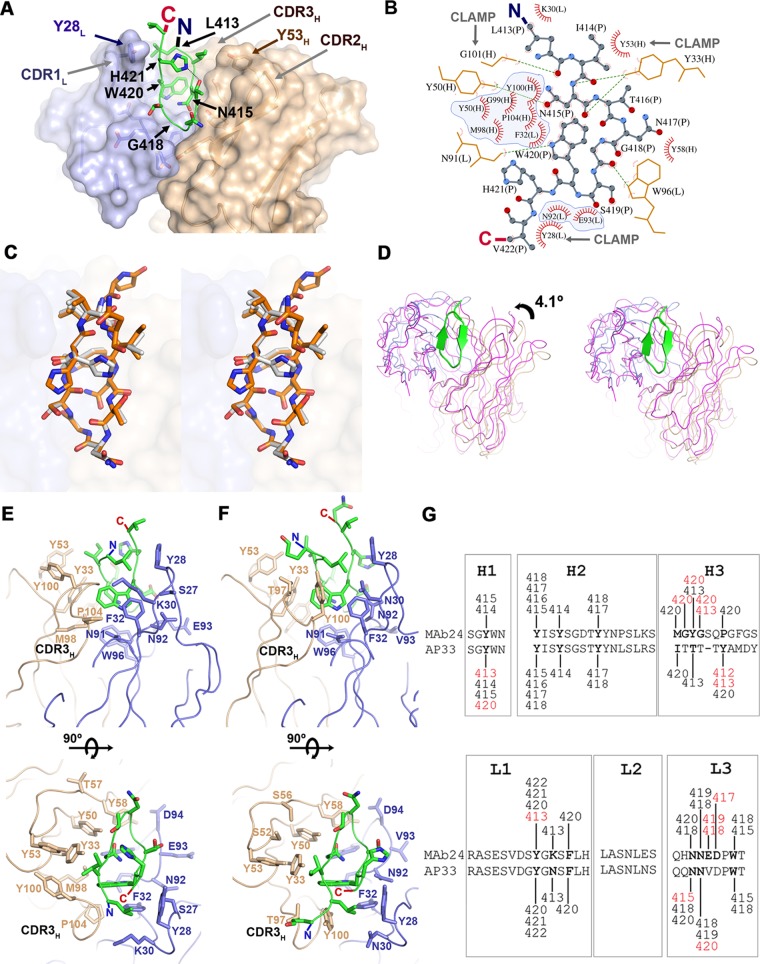
Recognition of E2 epitope I by MAb24. (A) Crystal structure of epitope I peptide E2_412-423_ (green) bound to the Fab of MAb24. The heavy and light chains are brown and light blue, respectively. Side chains of MAb24 within 4 Å of epitope I are shown as sticks. (B) Diagram of the interactions between epitope I (stick-and-ball representation) and MAb24. Hydrogen bonds are shown as green dotted lines, and Van der Waals contacts are shown as red arcs. Residues from epitope I and the light and the heavy chains are labeled P, L, and H, respectively. Intramolecular contacts are omitted. Residues contacting W420 of epitope I are shaded in blue. (C) Wall-eyed stereo view of epitope I peptides from MAb24 (white) and AP33 (orange; PDB code 4GAG) structures. (D) Wall-eyed stereo comparison of the orientation of MAb24 (blue/brown) and AP33 (magenta) with regard to epitope I. The respective epitope I chains were used for alignment of the complexes. (E, F) Orthogonal views of the paratopes of MAb24 (E) and AP33 (F). The representation scheme is the same as in panel A omitting the molecular surface. The CDR3s of the heavy chains bearing most of the differences between the antibodies are labeled CDR3_H_. (G) Comparison of contact residues between heavy- and light-chain CDRs with peptide between MAb24 and AP33. All contacts within 4 Å are shown. Differences between AP33 and MAb24 are red. The image shown was adapted from reference 23.

To understand the structural basis of MAb24 binding to E2, we determined the crystal structure of the antigen-binding fragment (Fab) of MAb24 in complex with a peptide from E2 epitope I corresponding to residues 412 to 423. When in complex with MAb24, epitope I is in a β-hairpin conformation (Fig. 5 and Table 3). This conformation differs markedly from the extended conformations observed with MAbs HC33.1 (25) and 3/11 (22) (Fig. 5 and 6A and B), which reinforces the notion that epitope 1 is highly conformationally flexible and yet antibodies to all known conformations possess broad neutralization activity against HCV isolates. Two other antibodies recognize epitope 1 in a similar hairpin conformation: human-derived HCV1 (24) and rodent-derived AP33 (16, 23, 26). The peptide adopts very similar conformations in all three structures with all-atom root mean square deviations (RMSDs) of 1.26 and 1.45 Å for AP33 and HCV1, respectively. However, recognition differs by the angles of approach. AP33 and MAb24 have similar angles of approach related by a 4.1° rotation around the peptide (Fig. 6C and D), while the orientation of HCV1 is drastically different (Fig. 5, 31.3° rotation). Thus, a different footprint on the intact E2 protein is anticipated. The RMSDs between C_alpha_ atoms of MAb24 Fab with AP33 and HCV1 are 0.65 and 3.49 Å for 418 and 387 equivalent residues, respectively.

**TABLE 3:** Data collection and refinement statistics^*a*^

Parameter	Value at:		
	Total resolution	Low resolution	High resolution
Resolution range (Å)	19.91–1.40	19.91–1.76	1.45–1.40
Completeness (%)	69.1	98.1	15
*I*/σ<*I*>	33.5	42.5	3.4
CC1/2 (%)	100	100	89.5
Redundancy	2	2	1.9
R_merge_	0.01	0.009	0.202
R_meas_	0.015	0.013	0.285
R_pim_	0.01	0.009	0.202
Refinement statistics			
Resolution (Å)	1.4		
No. of reflections	67,298		
*R*_work_/*R*_free_	0.171/0.190		
No. of atoms			
Protein	6,430		
Peptide	162		
Ligand/ion	154		
Water	447		
Avg B factors			
Protein	20.0		
Peptide	19.6		
Ligand/ion	44.0		
Water	34.1		
RMSDs			
Bond length (Å)	0.5		
Bond angle (°)	0.68		
MolProbity score (percentile)	99th		
Ramachandran plot (%)			
Favored regions	98.15		
Allowed regions	1.62		
Outliers	0.23		

a Statistical analysis was performed using XDS (42), BUSTER (46), CCP4 (52), and MolProbity (53).

Tryptophan 420 is a major contributor to the recognition of E2 for all epitope I-directed antibodies. In MAb24, this residue inserts itself deeply into the paratope and forms extensive hydrophobic interactions with both the heavy and light chains of the antibody (Fig. 6A and B). It also forms a hydrogen bond with the main-chain carbonyl of N91 of the MAb24 light chain. The heavy-chain CDR3 and light-chain CDR1 residue (Y28) form a clamp around the N and C termini of the peptide that appear to stabilize the hairpin conformation (Fig. 6B). Two other features may also favor a hairpin conformation: (i) Y33 and Y50 on the heavy chain, which make multiple hydrogen bonds with the main chain of the first strand of the peptide, and (ii) intrapeptide hydrogen bonding between the side chains of H421 and T416 that staples the two strands together. The requirement of H421 and T416 for MAb24 binding was confirmed in previous alanine substitution studies of the 412-to-423 region (7).

The intrapeptide interaction is not found in the high-resolution structure of AP33 epitope I (Fig. 6E and F, PDB code 4GAG), but it is present as an alternate conformation in a lower-resolution complex of AP33 with a slightly different peptide (PDB code 4GAJ) and in HCV1. Although the paratope surfaces of MAb24 and AP33 are very similar, their respective heavy-chain CDR3s differ in sequence and structure (Fig. 6E to G). In MAb24, interactions are mediated primarily by the main chain of CDR3_H_, while the side chain of Y100 forms the equivalent interacting surface in AP33. These differences in CDR3_H_ result in a tighter clamp in MAb24, including main-chain interactions by residue G101 (Fig. 6E and F).

### Structural basis of the impact of MAb24 escape mutations.

**FIG 7:**
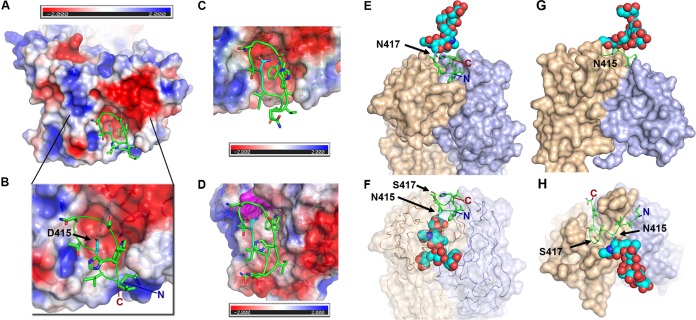
Structural impact of mutations at positions 415 and 417 on the recognition of epitope I. (A, B) Electrostatic surface of MAb24. The N415D mutation is modeled and shown in cyan. The peptide is shown as a green ribbon over the molecular surface of MAb24 colored according to its electrostatic potential. The red-white-blue ramp shows a scale of −2 to +2 kT. (C, D) Electrostatic surface of AP33 (C) and HCV1 (D) showing that N415 (cyan) faces W94 of the light chain, which is highlighted in magenta. (E) Epitope I of the MAb24-sensitive strain shown as a ribbon with a modeled basic glycan at position 417 (cyan) represented as spheres. (F) Epitope I of a MAb24-resistant strain bearing the N417S mutation modeled with a basic glycan at position 415 (cyan). (G, H) Orthogonal views of the N417S mutant in complex with HC33.1. A glycan is modeled at position N415 and is shown as cyan spheres.

The impact of the two mutations that enable MAb24 escape were modeled. Mutation N415D modeled in the hairpin conformation of epitope I places the aspartic acid side chain in a pocket of the paratope, which would bury 100% of its accessible surface. However, the paratope is largely electronegative and the introduction of the deprotonated D415 residue would create repulsive interactions, making it incompatible with binding (Fig. 7A and B). AP33 also has an electronegative surface facing residue 415 of E2 (Fig. 7C), while HCV1 presents a hydrophobic patch including the side chain of residue W94 in the light chain (Fig. 7D).

The second escape mutation, N417S, results in the shift of a glycan from position 417 to position 415 (Fig. 7E and F). The 415 site is predicted by *in silico* glycosylation, and biochemical evidence has been reported previously (30). The glycan at position N417, while not essential for the infectivity in genotype 2a viruses (31), is essential for viral entry in the context of genotype 1a HCV pseudoparticles (30). N415 glycosylation results in a steric clash with MAb24, regardless of the conformation of the glycan chain, since the base of the glycan directly faces the paratope (Fig. 7F). Thus, the glycan shift alone explains the ability of N417S mutants to escape MAb24 neutralization. In contrast, the presence of a glycan at position 415 does not present steric clashes that may impair ligand formation with antibodies recognizing epitope I in an extended conformation (Fig. 7G and H). The modeled glycan chain is positioned over a groove between the heavy and light chains of HC33.1, which may provide additional favorable interactions with the antibody.

**FIG 8:**
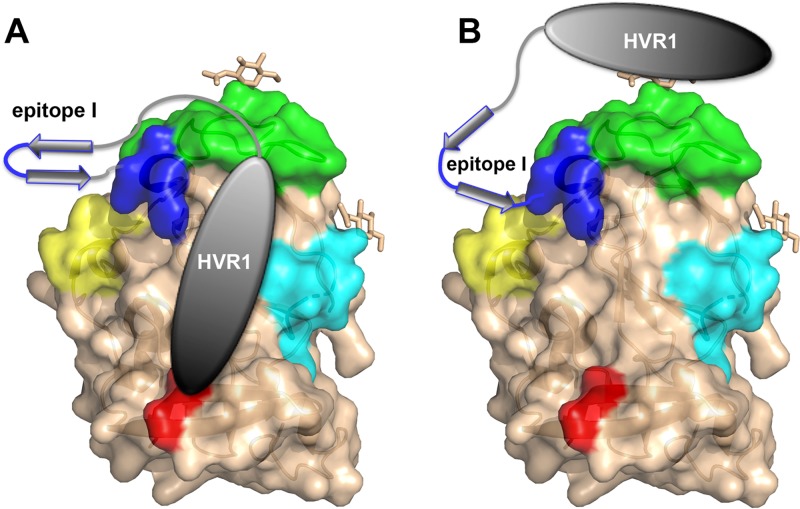
Models of the proposed interaction of epitope I, HVR1, and other antigenic regions. (A, B) Mutations in epitope I increase the sensitivity to MAbs targeting epitope I itself (blue; MAb HC33.1) and remote antigenic regions such as domain C (cyan; CBH-7), E2 components of AR5 (red; AR5A), domain D (yellow; HC84.1 and HC84.27), and the CD81 binding loop comprising part of domain B (green; CBH-5). The gray ellipse and arrows represent HVR1 and epitope I, respectively, in the MAb24-sensitive (left) and MAb-resistant (right) viral strains.

Overall, both mutations N417S and N415D render the virus more sensitive to HC33.1, which recognizes an extended conformation of epitope I, suggesting that either N415D or N417S may promote and/or stabilize an extended epitope I conformation, as depicted in Fig. 8. This conformational shift has an impact on antigenic regions distal from epitope I, enabling enhanced neutralization by human MAbs AR3C, CBH-5, and CBH-7 targeting the front face of E2, domain B, and domain C, respectively; MAbs HC84.27 and HC84.1 targeting domain D; and AR4A and AR5A targeting antigenic domains spanning the E1E2 complex.

## DISCUSSION

Direct-acting antivirals (DAAs) have an enormous potential to reduce the number of people infected with HCV, but major challenges remain regarding access to treatment, their high cost, the need to identify people who have active HCV infection, and the possibility of reinfection that will continue to drive the epidemic. The development of a vaccine that prevents primary infection and reinfection with HCV remains a priority and would substantially improve the effectiveness of DAAs and accelerate efforts to reach elimination targets set by the World Health Organization. The study of antibody responses to HCV envelope proteins both in natural infection and through vaccination of experimental animals has advanced our understanding of the mechanisms of neutralization and desirable antibody specificities that a vaccine should elicit.

The emerging model of glycoprotein E2 is one of a highly conformationally flexible protein that employs multiple immune evasion mechanisms to restrict the generation of bnAbs. The ability to delete all three variable regions and the C-terminal stalk (residues 643 to 711) without significant alteration of the overall conformational flexibility suggests that none of these regions contribute significantly to stabilization of the core domain of E2 (32, 33). Interestingly, over 62% of the structure of the E2 core domain structure was either in loops or was disordered, including residues 412 to 420 and 454 to 491, a loop at residues 585 to 596, and numerous N-linked glycans (8). In addition, the entire front layer of E2 containing the CD81 binding site and the majority of nAb epitopes is highly flexible (33). This level of conformational flexibility is analogous to that observed in HIV gp120 and represents a novel mechanism employed by highly variable viruses to suppress the production of nAbs by limiting the engagement with B cell receptors.

Multiple MAbs that target the 412-to-423 region have been isolated from humans naturally infected with HCV, as well as from animals vaccinated with E2-containing immunogens. While high-resolution structures of E2 in complex with these antibodies are not available, the structures of MAbs in complex with synthetic peptides have been solved and reveal that this region can adopt multiple conformations (7, 12, 15-21). In addition, electron microscopy reveals that one antibody to this region can have a 10 to 22° difference in the angle of approach to the E2 core domain (33). The conformational flexibility of this region is reflected by the absence of electron density in the two available E2 core domain structures. The ability to exist in multiple conformational states has been proposed to be an additional mechanism of immune evasion to maintain the subdominance of nAb epitopes in E2 (25, 33).

MAb24 was isolated by vaccinating mice with a minimized form of the E2 core domain lacking all three variable regions and truncated at residue 661 derived from genotype 1a prototype strain H77 lacking HVR1, HVR2, and the igVR. While MAb24 has the ability to neutralize all seven genotypes of HCV, the IC_50_s vary widely, even for identical sequences of the MAb24 epitope, which indicates a role for the regions surrounding epitope I in the 3D structure of E2 (7). Specifically, HVR1 preceding epitope I interacts with HVR2 and igVR, which are distant in the primary sequence and absent from existing crystal structures (7, 34). The ability of MAb24 to inhibit CD81 binding to Δ123 was 20-fold improved relative to that seen with intact E2, suggesting that the three variable regions alter the exposure or conformation of the MAb24 epitope, modulating epitope binding and subsequent CD81 blockade. Growth of genotype 2a viruses in the presence of increasing concentrations of MAb24 in addition to passaging of infected cells and cell-free virus resulted in the selection of escape variants that either contain a mutation in a contact residue at N415 to aspartic acid or contain a mutation that results in a shift of the N-linked glycan at position 417 to 415 (N417S). In the experiments performed here, we additionally detected viruses that contain N415D together with H386R in HVR1. Genotype 2a virus reverse engineered to contain N415D, N417S, or N415D/H386R is highly resistant to neutralization by MAb24. Acquisition of N415D results in a 6-fold reduction in the infectivity of genotype 2a viruses, but it is restored in viruses containing N415D/H386R, suggesting that H386R is a compensatory mutation that restores infectivity to WT levels.

The structure of residues 412 to 423 in complex with MAb24 reveals a β-hairpin conformation similar to that recognized by HCV1 and AP33. Mutation of N417 to Ser results in a glycan shift to N415 that sterically precludes binding of antibodies to the hairpin conformation of epitope I and explains how this mutation prevents neutralization by MAb24. Despite this ability to confer resistance to HCV1-like antibodies, examination of 7,486 sequences in the Los Alamos Database reveals that only 62 sequences contain a shift in glycosylation from 417 to 415, suggesting that this mutation is rare (<0.1%), whereas ∼5% of the sequences contain mutations at N415. This low frequency of mutations at positions 415 and 417 may result from a fitness cost for some genotypes. It is possible that mutations other than N415D, such as N415H/K/R/Y observed in naturally occurring variants, do not confer the same phenotype as that conferred by N415D. We attempted to introduce N415D and N417S mutations into the genotype 1a H77c isolate, but viruses were not infectious, suggesting that additional compensatory mutations may be required to support replication in cell culture (data not shown). However, these mutations were selected in genotype 1a H77 HCV-infected chimpanzees given 250 mg/kg HCV1 therapeutically, possibly suggesting that these mutations confer *in vivo* viral fitness (35).

For genotype 2a, the N417S mutation has no fitness cost and the N415D mutation is compensated for by the H386R mutation in HVR1. Another explanation for the low frequency of MAb24 escape mutations is that they do not prevent neutralization by antibodies recognizing an extended conformation of epitope I. Accordingly, modeling shows that glycan shifting from N417 to N415 is tolerated by HC33.1 where the new glycan extends outside the paratope. Indeed, a reverse-engineered genotype 2a virus containing N415D, N417S, or N415D/H386R was more easily neutralized by HC33.1 than the WT was, with a 5-, 3-, or 2-fold reduction in the amount of HC33.1 required to obtain 50% neutralization, respectively. It is possible that the introduction of N415D, or shifting of the glycan from N417 to N415, may favor an extended conformation, thus enhancing recognition by HC33.1-like antibodies in infection.

In addition, a more extensive analysis of neutralization by a broad range of other bnAbs to multiple antigenic regions of E2, including AS434 and AR3, as well as epitopes that involve a heterodimer of E1 and E2 (AR4 and AR5), reveals that MAb24 escape mutants are also more sensitive to neutralization by antibodies that recognize epitopes external to epitope I. This suggests that the N415D and N417S mutations may result in a global change in glycoprotein structure distal to epitope I, for example, through relocalization of HVR1, increasing the accessibility of other antigenic regions. Changes in HVR1 in the N415D mutant are further supported by the compensatory effect of mutation H386R in this region.

If the N415D and N417S mutations that elicit region 412-to-423-directed nAbs were selected in HCV-infected people, escape variants would be more susceptible to neutralization by other bnAbs and would therefore have a replicative disadvantage under immune pressure. These results suggest that vaccines that generate region 412-to-423-directed nAbs are highly desirable but should be accompanied by at least one other bnAb with specificity to an alternative bnAb epitope or an HC33.1-like specificity. Recently, we published the findings of a vaccination study performed with guinea pigs and Δ123, and reported broad and potent neutralization with antibody specificities detected toward the 412-to-423 region and AR3, suggesting that such antibody specificities can be induced by recombinant E2 vaccines (36). Our findings further advance efforts toward the production of prophylactic HCV vaccines that will be essential to support the elimination of hepatitis C as a public health threat.

## MATERIALS AND METHODS

### Cell culture and antibodies.

Human hepatocellular carcinoma cell line Huh7.5 was described previously (37), and cells were kindly provided by Charles Rice (Rockefeller University, USA). They were maintained in Dulbecco's modified Eagle medium (DMEM; Invitrogen) supplemented with 10% fetal bovine serum, 2 mM l-glutamine, 16.8 mM HEPES buffer, 40 μg/ml gentamicin, 1 μg/ml minocycline, and nonessential amino acids (DMF10NEA).

Human MAbs CBH-2, CBH-5, and CBH-7 were kindly provided by Steven Foung. Mouse anti-NS5A antibody 9E10 was a kind gift from Charles Rice. Human MAbs AR4A and AR5A were kindly provided by Mansun Law. The expression vectors for human MAbs HC84.1 and HC84.27 were described previously (7). Similarly, the heavy- and light-chain expression vectors for HCV1, HC33.1, and AR3C were constructed by synthesizing their variable heavy- and light-chain sequences according to their PDB information (4DGY, 4XVJ, and 4MWF, respectively) and subcloning them into vectors pcDNA3-tPA-LC and pcDNA3-tPA-HC. MAbs HCV1, HC33.1, AR3C, HC84.1, and HC84.27 were expressed in Freestyle 293F cells (Thermo Fisher Scientific, Australia) and harvested after 7 days of culture. MAb24 was produced by a mouse hybridoma cell line as previously described (7). The IgG was affinity purified with protein G Sepharose (GenScript, USA), eluted with 100 mM glycine (pH 2.8), and then neutralized with 1 M Tris-HCl (pH 8.0). The eluted IgG was dialyzed in phosphate-buffered saline (PBS), concentrated with Amicon Ultra Centrifugal Filter Units (10-kDa cutoff; Merck Millipore, Australia), and filtered via a 0.45-μm filter. Antibody concentration was determined by measuring the absorbance at 280 nm with a NanoDrop spectrophotometer (Thermo Fisher Scientific, Australia).

### Vectors and mutagenesis.

The DNA vector pJC1flag2 (p7-NS-GLUC2A) (JC1flag) was a kind gift from Charles Rice (38). The identified resistant mutations N415D and N417S were introduced into JC1flag DNA with the QuikChange II XL site-directed mutagenesis kit (Agilent, USA) in accordance with the manufacturer's instructions. The mutated and amplified constructs were digested with BsiWI and BglII and subcloned into similarly digested pJC1flag2. The DNA sequences were confirmed by using Applied Biosystems PRISM BigDye Terminator chemistry (version 3.1). The H386R/N415D double mutant was constructed by introducing the H386R mutation into a JC1flag construct containing N415D by the same procedure.

### Production of HCVcc.

Vector JC1flag DNA or JC1flag mutant DNA was linearized with XbaI and transcribed into RNA *in vitro* with the AmpliScribe T7 Flash Transcription kit (Epicentre, USA) before purification with the Qiagen RNeasy minikit (Qiagen, Germany). To produce HCVcc, viral RNA was transfected into Huh7.5 cells preseeded at 3.5 × 10^5^/well into six-well plates with DMRIE-C transfection reagent (Life Technologies, USA) in accordance with the manufacturer's recommendation. Alternatively, it was produced by electroporation of the RNA into Huh7.5 cells in T175 flasks. Three days after transfection, virus-containing cell culture supernatant was harvested and precleared before storage at −80°C.

### Virus passaging.

To investigate whether HCV can escape MAb24 neutralization, HCVcc produced by transfecting pJC1flag RNA was passaged in the presence of various concentrations of MAb24 in 24-well plates. The IC_50_, IC_70_, and IC_90_ of MAb24 against the starting HCVcc were first determined (0.245, 0.98, and 9.8 μg/ml, respectively). At time zero, HCVcc was incubated with 0, 0.245, 0.98, or 9.8 μg/ml MAb24 IgG for 1 h at 37°C before addition to a monolayer of naive Huh7.5 cells used to preseed 24-well plates at 4 × 10^4^/well. After 4 h, the medium was replaced with DMF10NEA containing MAb24 at the corresponding concentration. Cell culture supernatant was monitored for luciferase activity with the Renilla luciferase assay system (Promega, USA) and a FLUOstar OPTIMA reader fitted with luminescence optics (BMG Lab Technologies GmBH, Germany). This was repeated every 4 days. Alternatively, HCVcc was neutralized with MAb24 and passaged with infected cells at 3-day intervals. In addition to MAb24 neutralization at 37°C for 1 h and the addition of an extracellular HCVcc-MAb24 mixture to naive Huh7.5 cell monolayers (preseeded at 2.5 × 10^4^/well), the infected Huh7.5 cells from the previous passage were trypsinized and a fraction was added to the naive Huh7.5 cells. Renilla luciferase activity in the cell culture supernatant was as indicated. The concentration of MAb24 was increased to 2 times (passage 4), 5 times (passage 5), 10 times (passage 6), and 20 times (passage 7 onward) the corresponding starting concentration. The proportion of infected Huh7.5 cells added in each round of passage was reduced from 10% (passage 1) to 5% (passage 6) and 4% (passage 7 onward). The extracellular HCVcc and intracellular HCVcc (in infected cells) were collected at the end of each passage and stored at −80°C.

### Virus sequencing.

Viral RNA was extracted from the cell culture supernatant with the QIAamp Viral RNA minikit (Qiagen, USA). The E1E2 or E2 region was amplified with the SuperScript III one-step reverse transcription-PCR system with Platinum *Taq* High Fidelity (Invitrogen, USA). The resulting cDNA was cloned into the pGEM-T or pGEM-T Easy vector (Promega, USA), and clones were sequenced by using Applied Biosystems PRISM BigDye Terminator chemistry (version 3.1).

### Next-generation sequencing.

The primer design was as described in reference 39, in which a random eight-nucleotide tag (barcode) is inserted after the target sequence with the 5′ addition of a nonspecific sequence to allow amplification by PCR (5′-ACCTTGCAAGCACGCTCTGGCCTTGAANNNNNNNNCTTAAGC[barcode]CAGCGGTGGTCGAGTGCTGTTCAATAG-3′) corresponding to a specific primer ID. Primers were synthesized (Integrated DNA Technologies, USA). Accordingly, each cDNA template was synthesized with a unique primer ID (barcode tag), allowing accurate estimation of the viral species in the sample pool and the elimination of PCR artifacts in the analysis pipeline (40). Following cDNA synthesis, the reaction product was treated with RNase H (Invitrogen, USA) and purified with AMPure XP (Agencourt, Beckman Coulter).

The cDNA template was amplified by nested PCR with PrimeSTAR GXL DNA polymerase (Clontech, TaKaRa) in a 20-cycle first-round reaction, followed by a 25-cycle second-round nested reaction. The primers used to amplify the first-round product were 5′-TACAAGGACGACGACGACAAGGGC-3′ (sequence of the flag tag within JC1) and the 5′ portion of the primer used in the cDNA reaction mixture, 5′-ACCTTGCAAGCACGCTCTGGC-3′. The primers used to amplify the first-round template in a nested PCR were 5′-GCGCCTCACCAGCTTATTT-3′ (H77 reference position 1532) and 5′-CAAGCACGCTCTGGCCTTGAA-3′.

Amplicons were pooled in equimolar ratios and used as the input for library preparation with the Kapa Hyper Prep library kit in accordance with the manufacturer's protocol (Kapa Biosystems, Inc., Wilmington, MA, USA). Postquantitation of these library preparations was performed with the Kapa library quantitation kit (Kapa Biosystems, Inc., Wilmington, MA, USA), and they were sequenced on the MiSeq sequencer with the 600V3 kit (Illumina Inc., San Diego, CA) in accordance with the manufacturer's instructions. The data generated from the sequencing run were processed as follows. Sequence reads were aligned with the J6 reference sequence and the primer ID by pairwise alignment. Sequence reads that did not end with the primer ID sequence pattern were discarded. Reads were trimmed so that only sequences corresponding to the region of interest were included. A “majority rules” consensus was determined for each primer ID (minimum of three reads). The median number of unique primer IDs in the samples was 331 (interquartile range, 161 to 484). SNP reports were generated with the proprietary genome analysis tool VGAS.

### Virus infectivity and immunofluorescence.

Huh7.5 cells were used to preseed 24-well plates at a density of 6 × 10^4^/well on coverslips pretreated with 0.01% poly-l-lysine. Virus was diluted and allowed to infect cells for 4 h before replacement with fresh medium. Renilla luciferase activity was measured at 24, 48, and 72 h postinfection. To visualize the level of infectivity, infected cells were washed and fixed with ice-cold 100% methanol before blocking with 3% fetal bovine serum for 1 h at room temperature. Infected cells were detected with anti-NS5A antibody 9E10 at 1:3,000, followed by an Alexa Fluor 488-conjugated goat anti-mouse IgG secondary antibody at 1:3,000 (Invitrogen, USA). Cell nuclei were counterstained with propidium iodide for 5 min in the dark. Slides were mounted with coverslips and examined with an Olympus IX50 inverted microscope.

### Neutralization assays.

Huh7.5 cells were used to preseed 96-well plates at 6 × 10^3^/well. Antibodies were serially diluted with DMF10NEA before incubation with a constant amount of virus for 1 h in triplicate as described previously (36). The mixture was then added to the cells and incubated for 4 h before replacement with fresh medium. Cell culture supernatant was lysed after 72 h, and luciferase activity was measured. Assays were performed at least three times, except where indicated, and data were analyzed with Prism (v7.0) by using nonlinear regression analysis to determine the IC_50_s.

### Structural determination of MAb24 Fab in complex with a synthetic peptide spanning residues 412 to 423.

Briefly, the MAb24 IgG was digested in immobilized papain resin for 3 h at 37°C before purification on a protein A column and concentration and buffer exchange into PBS by using Amicon Ultra Centrifugal Filter Units (3-kDa cutoff; Millipore, USA). The cleavage of MAb24 IgG into Fab was confirmed by 10% SDS-PAGE and visualization by Coomassie blue staining.

### Protein crystallization and structure determination.

The purified Fab of MAb24 was concentrated to 6.5 mg · ml^−1^ and allowed to form a complex with the epitope I peptide (QLINTNGSWHVN, synthetically produced; GenScript, China) at a 1:5 molar ratio at 4°C for 2 h. Crystallization screening was performed with a Rigaku CrystalMation robotic system and Intelliplate 96-well crystallization plates. The reservoir volume was 50 μl, and sitting drops contained 100 nl of protein solution and 100 nl of precipitant. Hits were obtained in a PEG Ion HT crystallization screen (Hampton Research). Initial hits were optimized by the hanging-drop method with 2-μl drops and 1 ml of precipitant in each well. Small crystals were obtained in a drop containing 11.4 mg · ml^−1^ MAb24, 12.5% polyethylene glycol 3000 (PEG 3000), 100 mM NaCl, and 100 mM sodium phosphate dibasic/citric acid (pH 6.2). Addition of NaCl to the reservoir to a final concentration of 0.5 M was used to further increase the protein concentration in the crystallization drops, resulting in the formation of a hexagonal crystal within 48 h. The crystal was cryoprotected by brief immersion in a mixture of 20% glycerol, 12.5% PEG 3000, 0.5 M NaCl, and 100 mM sodium phosphate dibasic/citric acid (pH 6.2) before flash-cooling in liquid nitrogen.

Diffraction data were collected at a temperature of 100 K and an energy of 13,000 keV at the MX1 beamline of the Australian Synchrotron (41). Diffraction images were processed with *XDS* (42) and *POINTLESS* (43). The crystal belonged to space group I2 with unit cell parameters of a = 70.38 Å, b = 53.34 Å, c = 133.90 Å, α = γ = 90°, and β = 93.52°.

### Structure determination, refinement, and analysis.

The structure was determined by molecular replacement with Phaser (44) and the Phenix suite (45) by using the AP33 antibody (PDB code 4GAG) as an initial model (23). The model was refined with BUSTER (46) and built with Coot (47). LIGPLOT (48) was used to represent the van der Waals contacts and hydrogen bond interactions between the peptide and the antibodies. PyMOL (The PyMOL Molecular Graphics System, version 1.8; Schrödinger, LLC) was used to render images of the structure. The surface potential and electrostatics were calculated with the adaptive Poisson-Boltzmann solver (49). The binding surfaces were calculated by using the Proteins, Interfaces, Structures, and Assemblies server (50). Glycans were modeled by using the *in silico* glycosylation server GlyProt (51).

### Accession number(s).

Coordinates and diffraction data have been deposited in the Protein Data Bank under accession no. 5VXR.

## References

[1] Gower E, Estes C, Blach S, Razavi-Shearer K, Razavi H 2014 Global epidemiology and genotype distribution of the hepatitis C virus infection. J Hepatol 61:S45–S57. doi:10.1016/j.jhep.2014.07.027. 25086286

[2] Smith DB, Bukh J, Kuiken C, Muerhoff AS, Rice CM, Stapleton JT, Simmonds P 2014 Expanded classification of hepatitis C virus into 7 genotypes and 67 subtypes: updated criteria and genotype assignment web resource. Hepatology 59:318–327. doi:10.1002/hep.26744. 24115039 PMC4063340

[3] Ribeiro RM, Li H, Wang S, Stoddard MB, Learn GH, Korber BT, Bhattacharya T, Guedj J, Parrish EH, Hahn BH, Shaw GM, Perelson AS 2012 Quantifying the diversification of hepatitis C virus (HCV) during primary infection: estimates of the in vivo mutation rate. PLoS Pathog 8:e1002881. doi:10.1371/journal.ppat.1002881. 22927817 PMC3426522

[4] Petrovic D, Dempsey E, Doherty DG, Kelleher D, Long A 2012 Hepatitis C virus–T-cell responses and viral escape mutations. Eur J Immunol 42:17–26. doi:10.1002/eji.201141593. 22125159

[5] Michalak JP, Wychowski C, Choukhi A, Meunier JC, Ung S, Rice CM, Dubuisson J 1997 Characterization of truncated forms of hepatitis C virus glycoproteins. J Gen Virol 78:2299–2306. doi:10.1099/0022-1317-78-9-2299. 9292018

[6] McCaffrey K, Boo I, Poumbourios P, Drummer HE 2007 Expression and characterization of a minimal hepatitis C virus glycoprotein E2 core domain that retains CD81 binding. J Virol 81:9584–9590. doi:10.1128/JVI.02782-06. 17581991 PMC1951388

[7] Alhammad Y, Gu J, Boo I, Harrison D, McCaffrey K, Vietheer PT, Edwards S, Quinn C, Coulibaly F, Poumbourios P, Drummer HE 2015 Monoclonal antibodies directed toward the hepatitis C virus glycoprotein E2 detect antigenic differences modulated by the N-terminal hypervariable region 1 (HVR1), HVR2, and intergenotypic variable region. J Virol 89:12245–12261. doi:10.1128/JVI.02070-15. 26378182 PMC4665232

[8] Kong L, Giang E, Nieusma T, Kadam RU, Cogburn KE, Hua Y, Dai X, Stanfield RL, Burton DR, Ward AB, Wilson IA, Law M 2013 Hepatitis C virus E2 envelope glycoprotein core structure. Science 342:1090–1094. doi:10.1126/science.1243876. 24288331 PMC3954638

[9] Khan AG, Whidby J, Miller MT, Scarborough H, Zatorski AV, Cygan A, Price AA, Yost SA, Bohannon CD, Jacob J, Grakoui A, Marcotrigiano J 2014 Structure of the core ectodomain of the hepatitis C virus envelope glycoprotein 2. Nature 509:381–384. doi:10.1038/nature13117. 24553139 PMC4126800

[10] Giang E, Dorner M, Prentoe JC, Dreux M, Evans MJ, Bukh J, Rice CM, Ploss A, Burton DR, Law M 2012 Human broadly neutralizing antibodies to the envelope glycoprotein complex of hepatitis C virus. Proc Natl Acad Sci U S A 109:6205–6210. doi:10.1073/pnas.1114927109. 22492964 PMC3341081

[11] Tarr AW, Owsianka AM, Jayaraj D, Brown RJ, Hickling TP, Irving WL, Patel AH, Ball JK 2007 Determination of the human antibody response to the epitope defined by the hepatitis C virus-neutralizing monoclonal antibody AP33. J Gen Virol 88:2991–3001. doi:10.1099/vir.0.83065-0. 17947521

[12] Keck Z, Wang W, Wang Y, Lau P, Carlsen TH, Prentoe J, Xia J, Patel AH, Bukh J, Foung SK 2013 Cooperativity in virus neutralization by human monoclonal antibodies to two adjacent regions located at the amino terminus of hepatitis C virus E2 glycoprotein. J Virol 87:37–51. doi:10.1128/JVI.01941-12. 23097455 PMC3536422

[13] Owsianka AM, Timms JM, Tarr AW, Brown RJ, Hickling TP, Szwejk A, Bienkowska-Szewczyk K, Thomson BJ, Patel AH, Ball JK 2006 Identification of conserved residues in the E2 envelope glycoprotein of the hepatitis C virus that are critical for CD81 binding. J Virol 80:8695–8704. doi:10.1128/JVI.00271-06. 16912317 PMC1563869

[14] Boo I, Tewierek K, Douam F, Lavillette D, Poumbourios P, Drummer HE 2012 Distinct roles in folding, CD81 receptor binding and viral entry for conserved histidines of HCV glycoprotein E1 and E2. Biochem J 443:85–94. doi:10.1042/BJ20110868. 22240035

[15] Owsianka A, Clayton RF, Loomis-Price LD, McKeating JA, Patel AH 2001 Functional analysis of hepatitis C virus E2 glycoproteins and virus-like particles reveals structural dissimilarities between different forms of E2. J Gen Virol 82:1877–1883. doi:10.1099/0022-1317-82-8-1877. 11457993

[16] Owsianka A, Tarr AW, Juttla VS, Lavillette D, Bartosch B, Cosset FL, Ball JK, Patel AH 2005 Monoclonal antibody AP33 defines a broadly neutralizing epitope on the hepatitis C virus E2 envelope glycoprotein. J Virol 79:11095–11104. doi:10.1128/JVI.79.17.11095-11104.2005. 16103160 PMC1193588

[17] Tarr AW, Owsianka AM, Timms JM, McClure CP, Brown RJ, Hickling TP, Pietschmann T, Bartenschlager R, Patel AH, Ball JK 2006 Characterization of the hepatitis C virus E2 epitope defined by the broadly neutralizing monoclonal antibody AP33. Hepatology 43:592–601. doi:10.1002/hep.21088. 16496330

[18] Flint M, Maidens C, Loomis-Price LD, Shotton C, Dubuisson J, Monk P, Higginbottom A, Levy S, McKeating JA 1999 Characterization of hepatitis C virus E2 glycoprotein interaction with a putative cellular receptor, CD81. J Virol 73:6235–6244. 10400713 10.1128/jvi.73.8.6235-6244.1999 PMC112700

[19] Broering TJ, Garrity KA, Boatright NK, Sloan SE, Sandor F, Thomas WD Jr, Szabo G, Finberg RW, Ambrosino DM, Babcock GJ 2009 Identification and characterization of broadly neutralizing human monoclonal antibodies directed against the E2 envelope glycoprotein of hepatitis C virus. J Virol 83:12473–12482. doi:10.1128/JVI.01138-09. 19759151 PMC2786766

[20] Sabo MC, Luca VC, Prentoe J, Hopcraft SE, Blight KJ, Yi M, Lemon SM, Ball JK, Bukh J, Evans MJ, Fremont DH, Diamond MS 2011 Neutralizing monoclonal antibodies against hepatitis C virus E2 protein bind discontinuous epitopes and inhibit infection at a postattachment step. J Virol 85:7005–7019. doi:10.1128/JVI.00586-11. 21543495 PMC3126585

[21] Pantua H, Diao J, Ultsch M, Hazen M, Mathieu M, McCutcheon K, Takeda K, Date S, Cheung TK, Phung Q, Hass P, Arnott D, Hongo JA, Matthews DJ, Brown A, Patel AH, Kelley RF, Eigenbrot C, Kapadia SB 2013 Glycan shifting on hepatitis C virus (HCV) E2 glycoprotein is a mechanism for escape from broadly neutralizing antibodies. J Mol Biol 425:1899–1914. doi:10.1016/j.jmb.2013.02.025. 23458406

[22] Meola A, Tarr AW, England P, Meredith LW, McClure CP, Foung SK, McKeating JA, Ball JK, Rey FA, Krey T 2015 Structural flexibility of a conserved antigenic region in hepatitis C virus glycoprotein e2 recognized by broadly neutralizing antibodies. J Virol 89:2170–2181. doi:10.1128/JVI.02190-14. 25473061 PMC4338873

[23] Kong L, Giang E, Nieusma T, Robbins JB, Deller MC, Stanfield RL, Wilson IA, Law M 2012 Structure of hepatitis C virus envelope glycoprotein E2 antigenic site 412 to 423 in complex with antibody AP33. J Virol 86:13085–13088. doi:10.1128/JVI.01939-12. 22973046 PMC3497658

[24] Kong L, Giang E, Robbins JB, Stanfield RL, Burton DR, Wilson IA, Law M 2012 Structural basis of hepatitis C virus neutralization by broadly neutralizing antibody HCV1. Proc Natl Acad Sci U S A 109:9499–9504. doi:10.1073/pnas.1202924109. 22623528 PMC3386053

[25] Li Y, Pierce BG, Wang Q, Keck ZY, Fuerst TR, Foung SK, Mariuzza RA 2015 Structural basis for penetration of the glycan shield of hepatitis C virus E2 glycoprotein by a broadly neutralizing human antibody. J Biol Chem 290:10117–10125. doi:10.1074/jbc.M115.643528. 25737449 PMC4400327

[26] Potter JA, Owsianka AM, Jeffery N, Matthews DJ, Keck ZY, Lau P, Foung SK, Taylor GL, Patel AH 2012 Toward a hepatitis C virus vaccine: the structural basis of hepatitis C virus neutralization by AP33, a broadly neutralizing antibody. J Virol 86:12923–12932. doi:10.1128/JVI.02052-12. 22993159 PMC3497650

[27] Keck ZY, Xia J, Wang Y, Wang W, Krey T, Prentoe J, Carlsen T, Li AY, Patel AH, Lemon SM, Bukh J, Rey FA, Foung SK 2012 Human monoclonal antibodies to a novel cluster of conformational epitopes on HCV E2 with resistance to neutralization escape in a genotype 2a isolate. PLoS Pathog 8:e1002653. doi:10.1371/journal.ppat.1002653. 22511875 PMC3325216

[28] Owsianka AM, Tarr AW, Keck ZY, Li TK, Witteveldt J, Adair R, Foung SK, Ball JK, Patel AH 2008 Broadly neutralizing human monoclonal antibodies to the hepatitis C virus E2 glycoprotein. J Gen Virol 89:653–659. doi:10.1099/vir.0.83386-0. 18272755 PMC2885755

[29] Pierce BG, Keck ZY, Lau P, Fauvelle C, Gowthaman R, Baumert TF, Fuerst TR, Mariuzza RA, Foung SK 2016 Global mapping of antibody recognition of the hepatitis C virus E2 glycoprotein: implications for vaccine design. Proc Natl Acad Sci U S A 113:E6946–E6954. doi:10.1073/pnas.1614942113. PMC5111724 27791171

[30] Falkowska E, Kajumo F, Garcia E, Reinus J, Dragic T 2007 Hepatitis C virus envelope glycoprotein E2 glycans modulate entry, CD81 binding, and neutralization. J Virol 81:8072–8079. doi:10.1128/JVI.00459-07. 17507469 PMC1951298

[31] Helle F, Vieyres G, Elkrief L, Popescu CI, Wychowski C, Descamps V, Castelain S, Roingeard P, Duverlie G, Dubuisson J 2010 Role of N-linked glycans in the functions of hepatitis C virus envelope proteins incorporated into infectious virions. J Virol 84:11905–11915. doi:10.1128/JVI.01548-10. 20844034 PMC2977866

[32] Drummer HE, Poumbourios P 2004 Hepatitis C virus glycoprotein E2 contains a membrane-proximal heptad repeat sequence that is essential for E1E2 glycoprotein heterodimerization and viral entry. J Biol Chem 279:30066–30072. doi:10.1074/jbc.M405098200. 15136562

[33] Kong L, Lee DE, Kadam RU, Liu T, Giang E, Nieusma T, Garces F, Tzarum N, Woods VL Jr, Ward AB, Li S, Wilson IA, Law M 2016 Structural flexibility at a major conserved antibody target on hepatitis C virus E2 antigen. Proc Natl Acad Sci U S A 113:12768–12773. doi:10.1073/pnas.1609780113. PMC5111675 27791120

[34] Roccasecca R, Ansuini H, Vitelli A, Meola A, Scarselli E, Acali S, Pezzanera M, Ercole BB, McKeating J, Yagnik A, Lahm A, Tramontano A, Cortese R, Nicosia A 2003 Binding of the hepatitis C virus E2 glycoprotein to CD81 is strain specific and is modulated by a complex interplay between hypervariable regions 1 and 2. J Virol 77:1856–1867. doi:10.1128/JVI.77.3.1856-1867.2003. 12525620 PMC140892

[35] Morin TJ, Broering TJ, Leav BA, Blair BM, Rowley KJ, Boucher EN, Wang Y, Cheslock PS, Knauber M, Olsen DB, Ludmerer SW, Szabo G, Finberg RW, Purcell RH, Lanford RE, Ambrosino DM, Molrine DC, Babcock GJ 2012 Human monoclonal antibody HCV1 effectively prevents and treats HCV infection in chimpanzees. PLoS Pathog 8:e1002895. doi:10.1371/journal.ppat.1002895. 22952447 PMC3431327

[36] Vietheer PT, Boo I, Gu J, McCaffrey K, Edwards S, Owczarek C, Hardy MP, Fabri L, Center RJ, Poumbourios P, Drummer HE 2017 The core domain of hepatitis C virus glycoprotein E2 generates potent cross-neutralizing antibodies in guinea pigs. Hepatology 65:1117–1131. doi:10.1002/hep.28989. 27997681 PMC5408392

[37] Blight KJ, McKeating JA, Rice CM 2002 Highly permissive cell lines for subgenomic and genomic hepatitis C virus RNA replication. J Virol 76:13001–13014. doi:10.1128/JVI.76.24.13001-13014.2002. 12438626 PMC136668

[38] Marukian S, Jones CT, Andrus L, Evans MJ, Ritola KD, Charles ED, Rice CM, Dustin LB 2008 Cell culture-produced hepatitis C virus does not infect peripheral blood mononuclear cells. Hepatology 48:1843–1850. doi:10.1002/hep.22550. 19003912 PMC2592497

[39] Jabara CB, Jones CD, Roach J, Anderson JA, Swanstrom R 2011 Accurate sampling and deep sequencing of the HIV-1 protease gene using a Primer ID. Proc Natl Acad Sci U S A 108:20166–20171. doi:10.1073/pnas.1110064108. 22135472 PMC3250168

[40] Barnard R, Chopra A, James I, Blinco J, Watson MW, Jabara CB, Hazuda D, Lemon SM, Mallal S, Gaudieri S 2016 Primer ID ultra-deep sequencing reveals dynamics of drug resistance-associated variants in breakthrough hepatitis C viruses: relevance to treatment outcome and resistance screening. Antivir Ther 21:567–577. doi:10.3851/IMP3056. 27219495

[41] Cowieson NP, Aragao D, Clift M, Ericsson DJ, Gee C, Harrop SJ, Mudie N, Panjikar S, Price JR, Riboldi-Tunnicliffe A, Williamson R, Caradoc-Davies T 2015 MX1: a bending-magnet crystallography beamline serving both chemical and macromolecular crystallography communities at the Australian Synchrotron. J Synchrotron Radiat 22:187–190. doi:10.1107/S1600577514021717. 25537608 PMC4294030

[42] Kabsch W 2010 XDS. Acta Crystallogr D Biol Crystallogr 66:125–132. doi:10.1107/S0907444909047337. 20124692 PMC2815665

[43] Evans P 2006 Scaling and assessment of data quality. Acta Crystallogr D Biol Crystallogr 62:72–82. doi:10.1107/S0907444905036693. 16369096

[44] McCoy AJ, Grosse-Kunstleve RW, Adams PD, Winn MD, Storoni LC, Read RJ 2007 Phaser crystallographic software. J Appl Crystallogr 40:658–674. doi:10.1107/S0021889807021206. 19461840 PMC2483472

[45] Adams PD, Afonine PV, Bunkoczi G, Chen VB, Davis IW, Echols N, Headd JJ, Hung LW, Kapral GJ, Grosse-Kunstleve RW, McCoy AJ, Moriarty NW, Oeffner R, Read RJ, Richardson DC, Richardson JS, Terwilliger TC, Zwart PH 2010 PHENIX: a comprehensive Python-based system for macromolecular structure solution. Acta Crystallogr D Biol Crystallogr 66:213–221. doi:10.1107/S0907444909052925. 20124702 PMC2815670

[46] Bricogne G, Blanc E, Brandl M, Flensburg C, Keller P, Paciorek W, Roversi P, Sharff A, Smart OS, Vonrhein C, Womack TO 2016 BUSTER version 2.10.2. Global Phasing Ltd., Cambridge, United Kingdom.

[47] Emsley P, Lohkamp B, Scott WG, Cowtan K 2010 Features and development of Coot. Acta Crystallogr D Biol Crystallogr 66:486–501. doi:10.1107/S0907444910007493. 20383002 PMC2852313

[48] Wallace AC, Laskowski RA, Thornton JM 1995 LIGPLOT: a program to generate schematic diagrams of protein-ligand interactions. Protein Eng 8:127–134. doi:10.1093/protein/8.2.127. 7630882

[49] Baker NA, Sept D, Joseph S, Holst MJ, McCammon JA 2001 Electrostatics of nanosystems: application to microtubules and the ribosome. Proc Natl Acad Sci U S A 98:10037–10041. doi:10.1073/pnas.181342398. 11517324 PMC56910

[50] Krissinel E, Henrick K 2007 Inference of macromolecular assemblies from crystalline state. J Mol Biol 372:774–797. doi:10.1016/j.jmb.2007.05.022. 17681537

[51] Bohne-Lang A 2005 GlyProt: in silico glycosylation of proteins. Nucleic Acids Res 33:W214–W219. doi:10.1093/nar/gki385. 15980456 PMC1160146

[52] Winn MD, Ballard CC, Cowtan KD, Dodson EJ, Emsley P, Evans PR, Keegan RM, Krissinel EB, Leslie AGW, McCoy A, McNicholas SJ, Murshudov GN, Pannu NS, Potterton EA, Powell HR, Read RJ, Vagin A, Wilson KS 2011 Overview of the CCP4 suite and current developments. Acta Crystallogr D Biol Crystallogr 67:235–242. doi:10.1107/S0907444910045749. 21460441 PMC3069738

[53] Chen VB, Arendall WB, Headd JJ, Keedy DA, Immormino RM, Kapral GJ, Murray LW, Richardson JS, Richardson DC 2010 MolProbity: all-atom structure validation for macromolecular crystallography. Acta Crystallogr D Biol Crystallogr 66:12–21. doi:10.1107/S0907444909042073. 20057044 PMC2803126

